# Tumor-Associated Macrophages: Recent Insights and Therapies

**DOI:** 10.3389/fonc.2020.00188

**Published:** 2020-02-25

**Authors:** Jiawei Zhou, Ziwei Tang, Siyang Gao, Chunyu Li, Yiting Feng, Xikun Zhou

**Affiliations:** ^1^State Key Laboratory of Biotherapy and Cancer Center, West China Hospital, Sichuan University and Collaborative Innovation Center for Biotherapy, Chengdu, China; ^2^State Key Laboratory of Oral Diseases, National Clinical Research Center for Oral Diseases, West China College of Stomatology, Chinese Academy of Medical Sciences Research Unit of Oral Carcinogenesis and Management, Sichuan University, Chengdu, China

**Keywords:** macrophages, tumors, tumor-associated macrophages, immunity, immunity therapy

## Abstract

Macrophages, which have functions of engulfing and digesting foreign substances, can clear away harmful matter, including cellular debris and tumor cells. Based on the condition of the internal environment, circulating monocytes give rise to mature macrophages, and when they are recruited into the tumor microenvironment and in suitable conditions, they are converted into tumor-associated macrophages (TAMs). Generally, macrophages grow into two main groups called classically activated macrophages (M1) and alternatively activated macrophages (M2). M2 and a small fraction of M1 cells, also known as TAMs, not only lack the function of phagocytizing tumor cells but also help these tumor cells escape from being killed and help them spread to other tissues and organs. In this review, we introduce several mechanisms by which macrophages play a role in the immune regulation of tumor cells, including both killing factors and promoting effects. Furthermore, the targeted therapy for treating tumors based on macrophages is also referred to in our review. We confirm that further studies of macrophage-focused therapeutic strategies and their use in clinical practice are needed to verify their superior efficacy and potential in cancer treatment.

## Background

This review is based on the interaction of macrophages and tumor cells, and summarizes the origin, function, and classification of macrophages. Emphasis is placed on the dual role of macrophages in tumor cells and targeted therapy of related binding sites. The existing reviews about macrophages and the interaction with tumor cells are not a few, but the most are focused on one of the recognition mechanisms, specifically illustrating its molecular mechanism in detail. Nevertheless, based on the research findings in recent years, this review summarizes a variety of related mechanisms, sorts out and reintegration them to make them systematic. In the meanwhile, we also provide new ideas about tumor targeted therapy. Regarding tumor-targeted therapy, this review classifies them in treatment methods and sites to make the relevant treatment ideas clearer. There are still some methods that need further research, and this review explains and looks forward to the progress of the new step.

## Introduction

Macrophages, which are a type of white blood cells of the mononuclear phagocyte immune system, play vitally important roles in anti-infective immunity, the maintenance of tissue homeostasis, and the protection of our body through the functions of engulfing and digesting foreign substances (1, 2). Macrophages also clear away harmful matter, including cellular debris and tumor cells *in vivo*. Macrophages mediate non-specific defense (innate immunity) and help initiate specific defense mechanisms (adaptive immunity). In addition to stimulating the immune system, macrophages exert an immune modulatory impact by secreting various cytokines and activating the complement system, which may lead to inflammation.

Based on the conditions of the internal environment, such as the presence of chemokines, cytokines, and other factors secreted by tumor cells, mesenchymal cells, and immune cells, and the presence of local anoxia, inflammation, and high levels of lactic acid, the monocytic series in the blood are recruited to the tumor microenvironment and become tumor-associated macrophages (TAMs) (3, 4). Macrophages roughly develop into two main groups with different functions in immune defense and immune surveillance called classically activated macrophages (M1) and alternatively activated macrophages (M2), both of which can transform into each other with the changes in the internal environment.

Here, we introduce several kinds of mechanisms by which macrophages interact with tumor cells and kill them. Also, we compare these mechanisms with those by which TAMs play a role in promoting the development of tumor cells, in immune evasion and in immunosuppression. Therefore, based on macrophages differentiating into TAMs on cellular and molecular levels, our review shows several therapeutic targets for treating tumors caused by immunosuppression. In addition, we summarize some tumor therapy strategies at present aimed at macrophages, especially the theoretical basis and the feasibility of blocking the CD47-SIRPα pathway to treat tumors. In this way, engineered macrophages would play a significant role in suppressing tumors with potential clinical utility.

## A Simple Characterization of Macrophages

The origin of macrophages is still inconclusive, although it is currently universally believed that the major portion of macrophages is derived from monocytes in the peripheral blood circulation, as the mechanism has been clarified in some studies (5, 6). During the early stages of embryonic development, monocytes are recruited from marrow circulating blood and then travel to various tissues and organs via circulation, thus developing and differentiating into tissue-specific macrophages. Nevertheless, there are still some tissue-resident macrophages that are not derived from blood monocytes, such as alveolar macrophages in the lungs, microglia in the brain, and Kupffer cells in the liver, and the mechanisms of their origin, self-renewal, proliferation, and substitution have not been clarified as well (7). Recent studies confirmed the coexistence of tissue-resident macrophages proliferating *in situ* and those derived from blood monocytes in several tissues, including the lungs, spleen, and brain, and confirmed the phenotype and functions of these tissue-resident macrophages (8).

**Figure 1:**
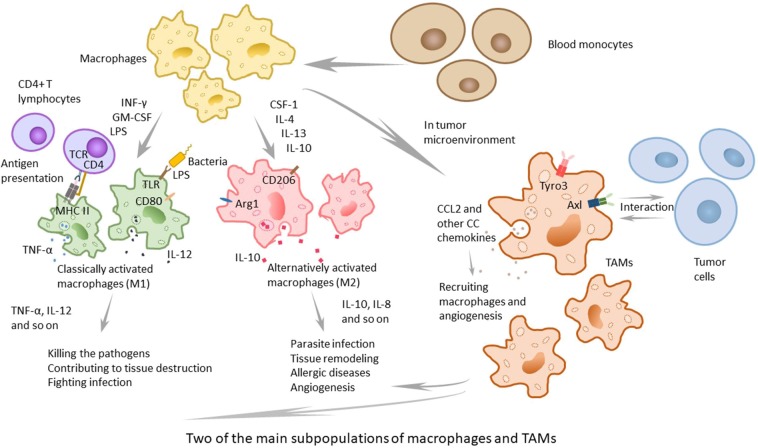
The two main subpopulations of macrophages and TAMs. Macrophages can be classified to several subpopulations, and the two main subpopulations are classically activated macrophages (M1) and alternatively activated macrophages (M2). M1 macrophages, active by IFNγ, GM-CSF, other cytokines and LPS, play an important role in human immune function and contribute to tissue destruction by producing proinflammatory cytokines with strong killing effects on pathogens. M2 macrophages, that can be active by CSF-1, IL-4, IL-13, IL-10, and other stimulation, participate in parasite infection, tissue remodeling, allergic diseases, and angiogenesis, and play an important role in above processes. TAMs, recruited in tumor microenvironment, are not a typical kind of macrophages and different from M1 or M2. They express special TAM receptors on membrane, and are interacted with tumor cells and play the dual role in tumor microenvironment.

In macrophage subpopulations, M1 macrophages, which produce proinflammatory cytokines with strong killing effects on pathogens invading the body, play an important role in human immune function and may contribute to tissue destruction. Cytokines, such as INF-γ, GM-CSF secreted by other immune cells and lipopolysaccharides (LPS) of the outer membrane of bacteria, can induce M1 macrophage activation (9, 10). M2 macrophages participate in parasite infection, tissue remodeling, allergic diseases, and angiogenesis, playing an important role in above processes. Previous studies have shown that CSF-1, IL-4, IL-13, IL-10, parasite infections, and other kinds of stimulation can lead macrophages to polarize to M2 macrophages (11, 12) (Figure 1). M1 and M2 are only two extreme descriptions of the polarization state of macrophages without covering a wide range of macrophage subpopulations (13). As an example, there are still CD169+ macrophages and TCR+ macrophages, and as is confirmed by present knowledge, in tumor-related studies, a large number of TAMs have been found in tumor-tissues (14). There is not much information about CD169+ macrophages and TCR+ macrophages, but present research has shown that they play certain roles in some respects. Some macrophages in the spleen, liver, bone marrow, lymph nodes, etc., express high levels of CD169 antigen on the surface. Relevant studies have failed to elucidate the relevant functions of CD169+ macrophages, but it is believed that CD169+ macrophages play a certain role in maintaining the homeostasis of the body, in immune regulation, and in immune tolerance (15–17). Concerning TCR+ macrophages, researchers discovered that TCR-αβ complex existed on 5–8% of neutrophils in the circulation (18), and Beham's group found that TCRβ gene rearrangement occurred in the early stage of bone marrow macrophages differentiation. TCR+ macrophages express chemokine (C-C motif) ligand 2 (CCL2) and have strong phagocytic ability, which is not the same as the functions of traditional macrophages (19).

## Tumor-Associated Macrophages, A Special Kind of Macrophages

The solid tumor consists of neoplastic cells and blood-born cells, including granulocytes, macrophages (up to 50%), and mast cells, as well as periphery cells—fibroblasts and epithelia (20, 21). Macrophages are recruited to the tumor site by the microenvironment, which produces cytokines. It has been proposed that the recruitment and differentiation progress are related to local anoxia, inflammation, and high levels of lactic acid. The CC chemokines, such as CCL2, CCL11, CCL16, and CCL21, which are major determinants of macrophage infiltration and angiogenesis, have been demonstrated to function in the cancer of breast, lung, esophagus, ovary and cervix, and CCL2 primarily contributes to the recruitment of macrophages (4, 22). Moreover, TAMs can produce CCL2, which means that they can recruit macrophages in turn. To some extent, TAMs can enlarge the recruitment of macrophages (23). Some studies and human diagnoses have demonstrated that the density of CCL2 is related to the quantity of TAMs, the tumor invasion and the clinical prognosis (Figure 1).

Involved in different microenvironments, macrophages acquire different specific phenotypes (3). The phenotypes of TAMs are plastic and regulated by the local microenvironment. Indeed, TAMs have been confirmed in recent studies to be present in large amounts in tumor tissues and to be significantly associated with tumor development progress. Strictly speaking, the division of macrophage types is complex. TAMs are not regarded as a classical subgroup of macrophages because these cells cannot be observed in the steady state but rather related to specific pathologic conditions, such as inflammation and tumors. There are some special receptor tyrosine kinases consisting of TAM receptor family, including Tyro3, Axl, and MerTK, and these receptors not only are of importance in interacting with tumor cells, but also play roles in macrophage polarization, efferocytosis and autoimmune disease (24). Active TAMs have several properties similar to M2. As a consequence, sometimes M2 macrophages are defined as TAMs in a narrow sense (14, 25). However, previous studies have shown that TAMs not only have the characteristics of M2 but also share M1 and M2 signature polarization. Therefore, the view that TAMs are equal to M2 is inaccurate (14). TAMs have profound effects on increases in angiogenesis, tumor invasion and the depression of immunity, as a result, TAMs can be taken into consideration in tumor immunotherapy (26, 27).

## The Dual Role of Tams in Tumor Microenvironment

Regarding the process of immune cells specifically recognizing and eliminating tumor cells, the mechanism is very complicated since various immune system components are involved, and macrophages are one of the most important members in these processes. TAMs are a key component of the leukocyte infiltrate that is seen broadly in various tumors. Examination of the roles of TAMs in tumor progression, in conjunction with investigations of other cells, has paved the way to eliciting new methods for tumor therapies. It's well-recognized that TAMs infiltrated in malignant metastatic cancers can promote tumor growth and metastasis, but that's not all, few kinds of macrophages subtypes can also have the antineoplastic activity.

### TAMs in Promoting Tumor Progression

#### Cytokines

Several studies have supported that TAMs can secrete chemokines and cytokines that promote the development of tumors, and studies on IL-6, IL-8, and IL-10 (typical examples) have made substantial progress in this respect.

##### IL-6

**Figure 2:**
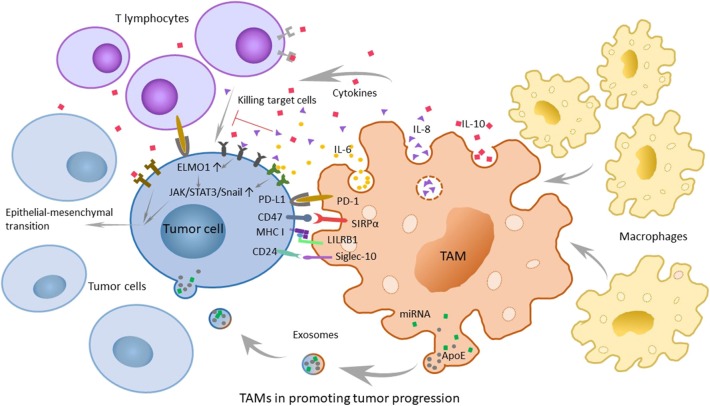
The role of tumor-associated macrophages (TAMs) in promoting tumor progression and related mechanisms. TAMs can secrete chemokines and cytokines that promote tumor development, such as IL-6, IL-8, and IL-10. Furthermore, various molecular mechanisms play a large role in immunosuppression. The PD-1/L1 signaling pathway promotes the possibility of tumor immune escape because it can inhibit the normal function of macrophages. The SIRPα/CD47 pathway is referred to as the “do-not-eat-me” signal, while tumor cells with CD47 expression can be recognized as self-normal cells. LILRB1/MHC class I component β2-microglobulin is also a significant mechanism of tumor escape. In breast cancer and ovarian cancer, CD 24 on tumor cells can promote immune escape through the interaction of Siglec-10. In addition, recent researchers have found that TAMs can promote the development of tumors through exosomes.

IL-6, secreted by tumor-associated endothelial cells and TAMs, is considered to increase the possibility of carcinogenesis and the developmental progress of malignant tumors by regulating the corresponding genes of the cell cycle, promoting tumor angiogenesis, aggravating local inflammation, and helping stem cell self-renewal. Because the major signaling pathway mediated by IL-6 is regulated by signal transducer and activator of transcription 3 (STAT3) phosphorylation and at the same time the epithelial-mesenchymal transition (EMT) is the main characteristic of tumor stem cells, the transcription factor Snail may have an important regulatory function (28). Therefore, researchers detected the expression of STAT3 phosphorylation and Snail in tumor cells interacted with TAMs and tumor-associated endothelial cells expressing or overexpressing B-cell lymphoma-2 (Bcl-2), which could promote the secretion of IL-6. And at the same time, they added a STAT3 suppressor to the group that overexpressed Bcl-2 and contained more IL-6. To obtain the results, the researchers tested the landmarks of the EMT. The results shows that IL-6 promotes STAT3 phosphorylation and the expression of Snail. When the phosphorylation of STAT3 was suppressed, the expression of Snail decreased simultaneously. The experimental results suggest that IL-6 may mediate the EMT by the janus kinase (JAK)/STAT3/Snail pathway (29). Another research also show s that IL-6 combined with IL-6R can activate STAT3 phosphorylation and lead to anti-apoptosis in tumors (30) (Figure 2).

##### IL-8

IL-8 is highly secreted by TAMs and serum IL-8 levels can correctly monitor and predict clinical benefit from immune checkpoint blockade. And experiments also showed that angiogenesis, tumor invasion, and the depression of immunity were more remarkable at higher levels of IL-8 (31, 32). Engulfment and cell motility 1 (ELMO1) is an evolutionarily conservative protein expressed in tumor cells that mainly mediates cell phagocytosis, migration, and morphological changes. Studies have shown that IL-8 can escalate tumor metastasis by upregulating the expression of ELMO1 in tumor cells (33). To a wide extent, the activation of the JAK2/STAT3/Snail pathway is considered to be another mechanism for the capability of IL-8 to promote carcinogenesis. With the increase in exogenous IL-8, the expression of p-JAK2, p-STAT3, and Snail shows extreme improvement. Hence, it is reasonable to speculate that IL-8 can promote EMT via the JAK2/STAT3/Snail pathway (34) (Figure 2). In inflammatory breast cancer (IBC), IL-8 and the growth-related oncogene (GRO) chemokines that activate STAT3 are strongly expressed, with monocytes recruitment and high-level expression of macrophage polarizing factors, promoting macrophages recruitment and transformation into M2, causing the highly infiltration. The highly infiltration macrophages also secrete high levels of IL-8 and GRO chemokines, resulting in a feed-forward chemokine loop that further drives the EMT of IBC (35).

##### IL-10

In the tumor microenvironment, TAMs secrete cytokines such as IL-10, transforming growth factor-β (TGF-β) and inflammatory mediators, including prostaglandin E2 (PGE2) and matrix metalloproteinase-7 (MMP-7), to inhibit the normal process of antigen-presenting, which makes T cells lose their competence in recognizing and even killing tumor cells. It is convinced that IL-10 family cytokines play an essential role during infection and inflammation to maintain tissue homeostasis, through upregulation of innate immunity, restriction of excessive inflammatory responses, and promotion of tissue repairing mechanisms (36). During chronic inflammation, toll-like receptor 4 (TLR4) can stimulate M2 to secrete the cytokine IL-10 (37). Moreover, the activation of TLR4 signaling by lipopolysaccharide profoundly increased the EMT in pancreatic cancer cells (Figure 2) and IL-10 increases cancerous the expression of inhibitor of PP2A (CIP2A) via the phosphatidylinositide 3-kinases (PI3K) signaling pathway and promotes tumor aggressiveness in lung adenocarcinoma (38, 39). Additionally, the researchers have found a positive correlation between IL-10 levels in serum and tumor progression, which shows that IL-10 has an important influence on promoting the development of tumors (40).

#### Immunosuppressive Receptors and Ligands

##### PD-1/PD-L1 signaling

Programmed cell death protein (PD-1) is a significant molecule in immunosuppression and belongs to the CD28 superfamily. It is of great importance to consider PD-1 as a target for immune regulation to fight tumors, for anti-infection, for autoimmune diseases and for organ transplantation survival. Its ligand, programmed cell death-ligand 1 (PD-L1), is the first type of transmembrane protein of 40 kDa. When the body is in a healthy condition, PD-L1 is expressed in antigen-presenting cells, which are combined with PD-1 carried by T cells, and the combination with PD-1 indicates that T cells will not launch an attack (41). However, just as tumor cells know the cipher sent to PD-1, PD-L1 can sometimes be expressed on the surface of tumor cells through poorly characterized oncogenic signaling pathways (42). T effector cells make a judgement that tumor cells are part of the “self”; thus, they are unable to kill the shrewd invaders. And in the meanwhile, PD-1 is also expressed on TAMs (43). The PD-1/L1 signaling pathway promotes the possibility of tumor immune escape because it can limit the functions of T effector cells, natural killer (NK) cells, dendritic cells, TAMs, and so on, such as suppressing activation, proliferation and cytokine expression effects on T cells and inhibiting the phagocytosis of TAMs (44) (Figure 2).

##### CD47-SIRPα signaling

**Figure 3:**
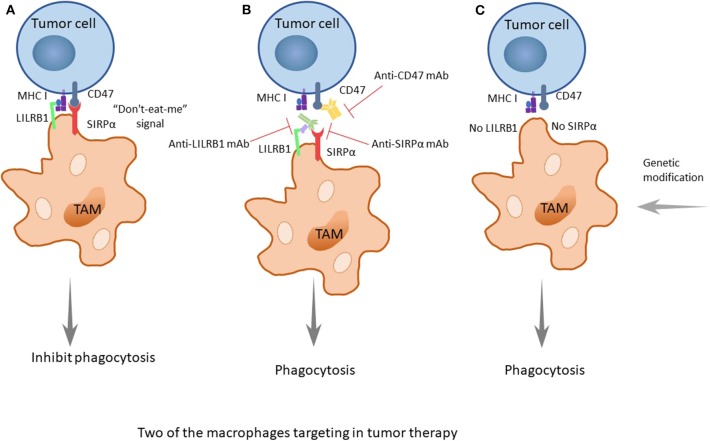
The two important treatment strategies targeting SIRPα/CD47 and LILRB1/MHC I. **(A)** CD47 is identified as a marker of self, or as a signal of “do-not-eat-me,” when the CD47 molecule of tumor cells is combined with its receptor, SIRPα, which is expressed on TAMs, and when MHC I on tumors is recognized by LILRB1 on TAMs; the signals will inhibit the phagocytosis of macrophages, promoting the occurrence, and development of tumors. **(B)** When using drugs such as monoclonal antibodies (anti-CD47 mAb, anti-SIRPα mAb, or anti-LILRB1 mAb), the recognition pathways are blocked, and the phagocytosis of macrophages is enhanced. Furthermore, the better treatment effect occurs when both pathways are blocked. **(C)** As it was discovered earlier that knocking out LILRB1 genes can cause macrophages to kill tumor cells, it could be predicted that more genetic modification of macrophages, such as making the SIRPα gene silent to suppress its expression, can achieve a similar effect.

The cluster of differentiation 47 (CD47) molecule is a membrane protein widely distributed on membrane surfaces of various cells, including tumor cells. Its corresponding ligand, signal regulatory protein alpha (SIRPα), is a membrane protein mainly expressed on macrophages and bone marrow cells, informing a typical immunoreceptor tyrosine-based inhibitory motif (ITIM). The interaction between the NH2 terminal domain of the ITIM motif and the single domain of CD47 can phosphorylate the ITIM motif, recruit the cytosolic tyrosine phosphatase SHP-1 or SHP-2 and activate it. As a consequence, this interaction can dephosphorylate multiple substrates and regulate downstream signaling pathways, ultimately inhibiting the phagocytosis of macrophages to normal cells. Therefore, CD47 is often referred to as the “do-not-eat-me” signal (45) (Figures 2, 3A).

The combination of these two molecules can produce a variety of physiological functions, and there is a balance between the two molecules. When the expression level of CD47 on the cell surface increases, the balance is upset, because CD47 sends out a “do-not-eat-me” signal to inhibit the phagocytosis of the tumor cells, promoting the occurrence, and development of tumors.

##### MHC class I component β2-microglobulin/LILRB1 signaling

Researchers have found that there are still some tumor cells escaping from the phagocytosis of macrophages after inhibiting the CD47 molecule. Recently, Weissman and colleagues have found that there is another recognition mechanism between tumor cells and macrophages that protects tumor cells from the phagocytosis of macrophages (46). The signaling molecule is the major histocompatibility complex (MHC) class I component β2-microglobulin on the surface of tumor cells (Figures 2, 3A). When the molecule is blocked or negative expression, macrophages can be awakened *in vivo* to enhance phagocytosis and eliminate tumor cells, extending the survival time of tumor-bearing mice by 70%. In addition, when researchers knockout the receptor leukocyte immunoglobulin like receptor subfamily B member 1 (LILRB1) on the surface of macrophages recognized by MHC class I molecules, macrophages change from promoting tumor growth to inhibiting tumor growth. Current studies have shown that the inhibition of the LILRB1 protein with the simultaneous administration of anti-CD47 monoclonal antibodies can significantly increase the phagocytosis and kill capacity of macrophages on tumor cells (Figure 3B), and the inhibition of LILRB1 does not damage normal tissue cells *in vivo*. Nevertheless, this mechanism needs further researches and clinical experiments (46).

##### CD24-siglec-10 signaling

Regarding the “do-not-eat-me” signal mentioned above, in the study of the magnitude and durability of the response to these agents such as monoclonal antibodies, the researchers found that there were still unclear escape signals. In breast cancer and ovarian cancer, Irving L, and his colleagues found that CD 24 was a dominant innate immune checkpoint and a promising target for tumor immunotherapy. They demonstrated that tumors expressing CD24 could promote immune escape through the interaction of inhibitory receptor sialic-acid-binding Ig-like lectin 10 (Siglec-10), which was expressed on TAMs. Further studies have shown that in addition to breast cancer and ovarian cancer, other tumors can also overexpress CD24, while TAMs express high levels of Siglec-10 (Figure 2). Blocking the interaction of CD 24 and Siglec-10 with monoclonal antibodies, or ablating the genes of CD 24 or Siglec-10, can both enhance the phagocytic function of TAMs to all human tumors expressing CD 24. This finding deserves further study and it proposes a new approach to tumor immunotherapy (47).

#### Exosomes From TAMs

Exosomes are small cell vesicles originating from cells that carry genetic information (proteins, nucleic acids, etc.) and mediate the information transmission and exchange of material between cells, which can affect the functions of target cells. In malignancies, exosomes serve as important carriers for materials and information exchange in the tumor microenvironment and participate in the survival and outgrowth of cancer cells and the different stages of tumor metastasis, which can be used as targets for tumor immunotherapy (48, 49). Previous studies have focused on the secretion of soluble signaling molecules such as cytokines and chemokines (50), while the discovery of exosomes provides a new idea for the correlation study of tumor immunity.

Recently, researchers have discovered that TAMs characterized by an M2-polarized phenotype can promote the metastasis of gastric cancer cells through exosomes (51) (Figure 2). TAMs can deliver exosomes to tumor cells, which are rich in miRNA, lncRNA, and specific proteins that can contribute to tumor metastasis. Mass spectrometric analysis reveals that M2-derived exosomes are rich in apolipoprotein E (ApoE), which can activate the PI3K-AKT pathway in tumor cells and induce the EMT and cytoskeleton rearrangement of gastric cancer cells, thus enhancing their metastatic potential as a consequence (51). Coincidentally, another research group studying the resistance of pancreatic ductal adenocarcinoma (PDAC) to gemcitabine have found that the mechanism by which TAMs help gemcitabine resistance may be related to exosomes. Using a genetic mouse model of PDAC and electron microscopy analysis, they found that TAMs secrete vesicles, with selective internalization by tumor cells, which indicated that TAMs and tumor cells communicate with each other. Furthermore, these authors also proved that the sensitivity of PDAC cells to gemcitabine could be significantly reduced by these macrophage-derived exosomes (MDE), which was mediated by the transfer of miR-365 in MDE (52). These discoveries open a new door for the study of the interaction between macrophages and tumor cells, and in quite a few ways, prompt researchers in this field to think about and study relevant mechanisms in greater depth. Perhaps further studies will discover the effects of exosomes on other tumors and their mechanisms for promoting tumor development, which are of great importance in clinical treatment.

### Enhancing the Antineoplastic Activity

#### Macrophage-Mediated Programmed Cell Removal (PrCR)

Macrophage-mediated programmed cell removal plays an important role in tumor elimination and surveillance. The activation of TLR pathways in macrophages induces the activation of Bruton's tyrosine kinase (Btk) signaling pathway (53), which makes the cell surface calreticulin (CRT) in endoplasmic reticulum phosphorylated and dissociated. The dissociated CRT is expressed on the surface of macrophages and then forms the CRT/CD91/C1q compounds to target cancer cells for phagocytosis (54). The induction of PrCR by “eat-me” signals on tumor cells is antagonized by “do-not-eat-me” signals, which bind macrophages SIRPα to inhibit phagocytosis. Blocking CD47 on tumor cells will block “do-not-eat-me” signals. Therefore, the activation of TLR signaling pathways in macrophages can synergize with blocking CD47 of tumor cells to enhance PrCR.

#### Enhancing the Toxicity

Activated macrophages defend against tumors by directing tumor cytotoxicity and by secreting cytokines. Researchers enhance macrophage cytotoxicity through specificity to stimulate activation, such as by adding M-CSF and muramyl dipeptide (MDP) when macrophages are cultured *in vitro* to enhance macrophage cytotoxicity; by using the adoptive transfer treatment to achieve anti-tumor effects; or by using intravenous liposomes that load immune modulators to enhance the toxicity of macrophages. The molecules of microbial agents and pathogens can stimulate the antitumor cytotoxicity of macrophages, such as using bacilli calmette-guerin (BCG) in the treatment of bladder cancer, through stimulating macrophages to increase the cytotoxicity of macrophages to certain bladder cancer cell lines (55). In addition, there is evidence that the increased levels of IL-6, IL-12, and TNF in the urine of bladder cancer patients treated with BCG may be related to the enhancement of the function of macrophages. Sialyl-Tn (sTn) is a kind of glycan that controls synthesis by sialic acid transferase ST6GALNAC1 and is abnormally expressed in bladder cancer cells. The researchers established a bladder cancer cell lines that expressed sTn (MCRsTn) in the process of study where sTn participated in the BCG treatment of bladder cancer. These researchers found that the secretion of BCG could promote MCRsTn to secrete IL-6 and IL-8. These cytokines further stimulate macrophages to produce large amounts of IL-6, IL-1 and TNF-α to enhance the toxicity of macrophages to tumor cells (56).

#### Preventing the Diffusion and Metastasis of Cancers

In recent years, researchers have discovered sub-membranous lymphoid sinus macrophages (SCS macrophages), which can form a protective membrane around lymph nodes to prevent the growth and metastasis of tumors (57). Present studies have demonstrated that a potential way in which information transfer can occur between tumor cells and immune cells. Tumor-derived extracellular vesicles (tEVs), especially highly concentrated near lymph nodes, can leave the tumor tissue and migrate to the whole body. They are vital participators in this way (58). As has been found in some studies, tEVs can interact with SCS macrophages, which form a layer of cells in the fibrous capsule surrounding the lymph nodes, thus limiting the spread of tEVs, preventing the entry of tEVs into lymph nodes, and blocking the pathway that causes B cells to produce tumor-promoting growth substances, thereby inhibiting the migration and transformation of melanoma. The specific molecular mechanism remains to be elucidated (59). In this case, the protection of SCS macrophages against tumor growth can be considered in the treatment of tumors; with further development of this research, additional mechanisms and whether this mechanism exists in other tumors will be discovered, and this recommendation needs more clinical experiments and confirmation.

## Macrophages Targeting in Tumor Therapy

In recent years, tumor immunotherapy has been widely concerned and made remarkable progress. By adjusting the immune defense function of the body, tumor immunotherapy can transform immune cells or use various types of immune-active substances to achieve balance between immune system and tumors. CAR-T and PD-1/PD-L1 blockade therapy has achieved significant clinical efficacy. Macrophages, as the important members of tumor microenvironment, become potential hot spots for immunotherapy drug development because of their characters. Next, we will summarize various tumor immunotherapy strategies targeting macrophages and their application prospects.

### Macrophages Targeting Therapy (Table 1)

It has been known that the use of non-discriminatory medicine for the whole body in the treatment of tumors has many disadvantages, such as damaging the immune system and upsetting the equilibrium of the microenvironment or even the entire balance. Therefore, in seeking a treatment that damages the tumor only, one concern, the need for targeted therapy and modification of molecules in the expression pathways, has been present for a long time.

**Table 1:** Macrophages targeting therapies.

**Category**	**Substance**	**Target site**	**Mechanisms of action**
Inhibitor	Zoledronic acid	CCL2	Suppress the expression of CCL2
	Gefitinib	CCL5	Decrease the secretion of CCL5
	PLX3397	CSF1R	Inhibit the expression of CSF1R
	GW2580	CSF1	Inhibit the expression of CSF1
	Wortmannin	PI3K	Decrease serum cytokine levels by inhibiting PI3K
Monoclonal antibody or blocker	HAC	PD-L1	Block human PD-L1
	BMS-936558	PD-1	Block the interaction between PD-1 and PD-L1
	Hu5F9-G4	CD47	Bock CD47 that induces tumor-cell phagocytosis
	KWAR23	SIRPα	Combined with tumor-opsonizing antibodies to augment neutrophils and TAMs antitumor activity
	GHI/75	LILRB1	Bock the MHC I/LILRB1 signaling way
	Trabectedin	Macrophages	Block the immunosuppressive effect
	Immunomodulator linemode	Macrophages	Block the activity of macrophages in tumor angiogenesis
Biological response modifier	DNMTi 5-Azacytidine (AZA)	Macrophages	Regulate of macrophages polarization
	α-Difluoromethylornithine (DFMO)	Macrophages	Regulate of macrophages polarization
	Dual-inhibitor-loaded nanoparticles (DNTs)	M2 macrophages	Make M2 macrophages repolarize to active M1 macrophages and inhibit CSF1R and SHP-2

*In this table, we summarize the relevant macrophages targeting therapies mentioned in chapter 5 about anti-tumor cells. It divides the drugs into inhibitor, monoclonal antibody, or blocker and biological response modifier, and in each category, it contains substance, target site, and mechanisms of action of these drugs*.

#### CCL2 and CCL5

Stimulated by proinflammatory factors, such as IL-8 and TNF-α, a large secretion of CCL2 (also known as monocyte chemotactic protein-1, MCP-1) occurs by activated macrophages, monocytes and dendritic cells. In other words, the interaction between resident macrophages and newly recruited macrophages is bidirectional because resident TAMs conversely can recruit macrophages to deteriorate tumor metastasis. As a peritumoral function of TAMs, CCL2 is considered a promising target site to prevent the tissue from collecting TAMs (60). Recently, researchers have found that zoledronic acid, a diphosphate compound, can suppress the expression of CCL2/MCP-1, decreasing the number of recruited macrophages and performing an antitumoral function (61). A high concentration of CCL5 can also bring about the recruitment of TAMs by connecting with CCR2 on the surface of monocytes in some cases. Gefitinib, a tyrosine kinase inhibitor that can decrease the secretion of CCL5, inhibits the cross-talk between TAMs and prostate cancer cells, leading to the proliferation of the tumor cells and the inhibition of docetaxel activity (62).

#### Colony Stimulating Factor-1 (CSF-1)

Many studies on targeted therapy are based on a purposeful strategy of CSF1/CSF1R, that is, to focus on the recruitment of TAMs and the secretion of cytokines, tumor cells secrete CSF1 for the purpose of collecting TAMs by connecting CSF1 with CSF1R on macrophages. CSF1 is related to macrophage recruitment, differentiation and repolarization; thus, it is an effective way to target CSF1/CSF1R. As was shown in a previous study, the tyrosine kinase inhibitor PLX3397 was used for the treatment of melanoma in mouse models driven by BRAFV00E. It shows the ability to inhibit CSF1R, and through its inhibition of the CSF1R, it is currently used as the treatment of patients with glioblastoma, breast cancer, and other cancers in clinical. These researchers found that the number of TAMs was remarkably reduced and that the proportion of M2 also decreased (63). Similarly, in MMTV-Neu transgenic mice, inhibiting the CSF1/CSF1R pathway by a CSF1 inhibitor named GW2580 led to a noticeable decrease of TAMs infiltration in tumor tissue (64). Another study showed that with the assistance of inhibitor PLX3397 or a monoclonal antibody of CSF1, CSF1-deficient mice showed specific changes, such as the decrease number of TAMs (65). It is now generally believed that the loss of the CSF1/CSF1R signal possesses the ability to give absolute control for consuming M2 macrophages, contrary to the uninfluential M1 macrophages (66).

#### Related Kinase Signaling Blocking

According to the description above, IL-10 promotes the growth and transfer of tumors by increasing CIP2A expression via the PI3K signaling pathway. Studies show that IL-10 secreted in E6-positive lung cancer cells is regulated by the phosphorylation of cAMP response element binding protein (CREB) via the pathway, and the feedback of IL-10-CIP2A-phosphorylated-CREB is likely to affect the progression of tumors. One of the targeted therapies uses specific inhibitors, such as wortmannin or LY294002 (PI3K inhibitors), to block the signaling transduction pathway. Wortmannin, a commonly used cell biology reagent, has been previously used to suppress DNA repair, receptor-mediated endocytosis and cell proliferation (67). Wortmannin has been confirmed to be effective in decreasing serum cytokine levels by inhibiting PI3K/Akt, which may suppress tumor invasiveness. In recent research, Halaby et al. have discovered serine-threonine kinase general control nonderepressible 2 (GCN2) is important to maturation and polarization of macrophages and myeloid-derived suppressor cells (MDSCs) by promoting translation of the transcription factor CREB-2/activating transcription factor 4 (ATF4). Therefore, they blocked the GCN2 signaling by targeting Atf4 with small interfering RNA knockdown, and found that tumor growth was reduced as a consequence. This finding demonstrates blocking GCN2 signaling can promote anti-tumor immunity (68).

#### PD-1/PD-L1 Signaling Blocking

One study treated immunocompromised mice with either a PD-L1 blocker (HAC, an engineered small protein which can block human PD-L1) or a PD-1 blocker (anti-mouse PD-1 antibody). The results show that both murine and human TAMs express high levels of PD-1, and the level of PD-1 increases gradually with the development of tumors. After PD-1/PD-L1 suppression by inhibitors, the phagocytosis function of TAMs improves, killing tumor cells. In addition, it is likely that PD-1/PD-L1 therapies interact with anti-CD47 in the context of macrophage-mediated immunotherapy, and the combination therapy trends toward increasing the survival rate more than monotherapy (43). According to the PD-1/PD-L1 recognition mechanism, many PD-1 monoclonal antibodies, such as BMS-936558, have been approved by the FDA for use in clinic and have achieved great efficacy in the treatment of certain advanced malignant tumors, although PD-1 inhibitors have a curative effect only on a small proportion of cancer patients (69).

#### Monoclonal Antibodies and Inhibitors

Immune escape is one of the most important mechanisms of tumor establishment and diffusion. Currently, the most widely used tumor immunotherapy is monoclonal antibodies. Monoclonal antibodies can block multiple pathways involved in TAMs and tumors recognition, disrupting tumors escape pathways and thus acting as antitumor agents. After discovering the CD47-SIRPα recognition mechanism of tumor cells and macrophages, researchers used an anti-CD47 monoclonal antibody to carry out *in vivo* experiments on tumor-bearing mice, and found the antibody can block the CD47-SIRPα pathway to interdict the signal of anti-phagocytosis (Figure 3B). This antibody shows targeting to tumor cells, which increases the macrophage phagocytosis of tumor cells and at the same time, does not affect normal cells (70). The CD47 molecule is also expressed on the surface of normal cells, and the anti-CD47 mAb triggers a strong self-reaction (71–73). Current researches have found that anti-CD47 monoclonal antibodies mainly induce transient anemia and mild neutrophil reduction as well as no other obvious adverse effects or the occurrence of autoimmune diseases (74, 75). However, Hu5F9-G4, an anti-CD47 monoclonal antibody, selectively eliminates malignant cells that express CD47 and not normal cells (76). As is mentioned in a recent study, glutaminyl-peptide cyclotransferase-like protein (QPCTL) is identified as a new target to interfere with the CD47 pathway and promotes the efficacy of antibody therapy of cancer (77). Recently, Arely and colleagues switched to an anti-SIRPα monoclonal antibody in the study and blocked this mechanism to enhance the tumor phagocytosis of macrophages; the effect was better than that in previous experiments (78), and another research team found the anti-human SIRPα antibody, KWAR23, could significantly promote the anti-tumor activity of neutrophils and TAMs when it was in combination with the tumor-opsonizing antibody rituximab (79) (Figure 3B).

Though the mechanism of the anti-CD47 antibody is not yet clear, the possible pathways are as follows: preventing the combination of CD47 on tumor cells and SIRPα on macrophages to activate phagocytosis, promoting the cytotoxic effect of antibody dependence and complement dependence based on Fc, directly inducing apoptosis to tumor cells, or stimulating the phagocytosis of dendritic cells to tumor cells. Additionally, it is likely the combined result of several mechanisms mentioned above (45). Because the overexpression of CD47 in myeloid leukemia cells prevents macrophages from clearing tumor cells, the survival rate of tumor cells increases. Taken together, these findings provide a rational basis for targeting the interaction of CD47-SIRPα in cancer, particularly to enhance the efficiency of antibody therapy in cancer. Similarly, drugs of another recognition mechanism, LILRB1/MHC class I, such as the LILRB1 monoclonal antibody GHI/75 are still in the clinical trial stage, and no obvious damage to the human body has been found for the time being (46) (Figure 3B). These drugs have clear targets and few adverse reactions, providing a theoretical basis and good prospects for clinical application. In addition, in some studies, macrophage-mediated antibody-dependent cell phagocytosis (ADCP) has been elucidated, which needs more experiments to study its mechanism (80).

#### Regulation of Macrophages Polarization

In recent years, using molecular targeted drugs to treat hepatocellular carcinoma has led to new breakthroughs with deep researches in the molecular biology of liver cancer. The treatment strategies for macrophages in the microenvironment of hepatocellular carcinoma include promoting M2 macrophages to transform into M1 macrophages (81) and blocking the immunosuppressive effect. Trabectedin is a targeted drug for macrophages and is used to treat soft tissue sarcomas. This drug is a marine bioactive extract that is toxic to macrophages. Other potential drugs, such as the immunomodulator linemode, can block the activity of macrophages in tumor angiogenesis. The CCL2 antibody can reduce the aggregation of macrophages as a potential treatment. C-Fms is a CSF receptor that regulates the function of macrophages. Clinical research is conducted using many drugs and these drug combinations may affect the interaction of C-Fms with other immune cells, change macrophage phenotypes and change the microenvironment that maintains M2 macrophages.

Combination therapy can also be put in to use in the treatment of cancer. A recent study held by Travers found that DNMTi 5-Azacytidine (AZA) and α-difluoromethylornithine (DFMO) in combination could significantly improve survival, reduce tumor burden, and then they combined therapy in a mouse model of ovarian cancer with normal immune function. The survival rate significantly decreased, more than that the two drugs were used alone. Significant reduction in M2-polarized macrophages and increased number of tumor-killing M1 macrophages in combination therapy suggest that combination therapy can alter macrophage polarization in the tumor microenvironment, recruit M1 macrophages and prolong survival period (82). This type of tumor suppression treatment will have great prospects for clinical application.

In addition, a new study by Ashish Kulkarni and his colleagues have reported that self-assembled dual-inhibitor-loaded nanoparticles (DNTs) target M2 macrophages and make M2 macrophages repolarize to active M1 macrophages. In the meanwhile, this drug simultaneously inhibits CSF1R and SHP-2 signaling pathways. This research provides an idea for anti-tumor therapy of macrophages and DNTs has good perspective potential for individual drug treatments (83).

### Engineering Macrophages

#### Macrophage Gene Modification

In the discovery of a new mechanism for the recognition between macrophages and tumor cells, the MHC class I component β2-microglobulin/LILRB1 protein, researchers used the gene modification of macrophages to knock out the gene for the LILRB1 protein and downregulated its expression on the membrane surface, allowing the macrophages to transform from the state of promoting the growth of tumor cells to eliminating the tumor cells (Figure 3C). While inhibiting the receptor with the simultaneous administration of anti-CD47 monoclonal antibodies, the phagocytosis and killing capacity of macrophages on tumor cells is significantly increased (46) (Figure 3B). In recent research, researchers have found that CD 24 expressed on tumor cells is a dominant innate immune checkpoint, and can promote the escape of tumors with the interaction of Siglec-10 on TAMs. Ablating the genes of CD 24 or Siglec-10 has been demonstrated an effective way to enhance the phagocytic function of TAMs (47). At present, due to the convenience of clinical application and cost issues, macrophage gene modification is not as frequently used, as it is only at the research stage; however, in future, with the development of the technology, gene therapy, especially the progress of genetic engineering, will have better prospect because of its stability and longevity. When the technology is mature and applied on a large scale, tumor treatment and precision medicine will take a new step.

#### iSNAPS Smart Protein Molecules

A group of researchers designed a smart protein called the integrated sensing and activating proteins (iSNAPS) protein, which could reprogram white blood cells and ignore the self-defense signaling mechanisms on which tumor cells rely for survival and spreading *in vivo*. The emergence of this protein will present new approaches and ideas for the editing of immune cells. The iSNAPS protein is inserted into macrophages in the study, and it reconnects macrophages, covering the escape signals recognized by the tumor cells and interpreting them as phagocytic signals. In addition, its rapid response and strong lethality can significantly enhance the ability of macrophages to divide, phagocytose, and kill tumor cells rapidly (84).

The design principle of this intelligent protein molecule can also be used to redesign other immune cells for cancer treatment. At present, the team plans to test iSNAPS in mice and may study its application in other areas (84). This protein may influence not only tumor treatment but also other diseases and self-regulation, and further research is needed.

## Conclusions

This review introduces the origin, classification and immune function of macrophages and further explores the mechanisms of the participation of macrophages in tumor microenvironment. We focus on the killing effect and mechanisms of macrophages on tumors, while tumor promoting factors such as IL-6, IL-8, IL-10, TLR4 are briefly introduced as well (29, 37, 40, 85). Based on existing research, we discuss the molecular mechanisms of the interaction between macrophages and tumor cells, not only the chemokines and cytokines but also some recognition mechanisms including. For instance, the promoting pathways of PD-1/PD-L1, SIRPα/CD47, and LILRB1/MHC I (41, 46, 86) and the killing factors such as PrCR (54) are presented. In addition, based on existing researches, we summarize a new pathway by which TAMs can promote the development of tumors through exosomes. The pathway has been found and may exist in certain kinds of tumors, which opens a new door for the study of tumor immunity (51, 52). Moreover, several types of treatments, such as inhibiting M2 macrophages to promote the growth of tumor cells, motivating the transition of M2 macrophages to M1 macrophages, enhancing macrophage phagocytosis of tumors and reinforcing the role of macrophages in preventing tumor growth and metastasis, suggest that macrophages can participate in tumor cells immune regulation through various molecular mechanisms and should be given more attention.

At present, with the development of precision medicine, the therapeutic direction of tumors has gradually turned to targeted therapy because non-discriminatory medicine for the whole body during the treatment of tumors has many disadvantages. With tumor immunity becoming a popular research direction, increasing researches has been conducted to overcome the unresolved issues in traditional tumor treatment, but this area of research has been very limited in terms of adaptive immunity until recent years. As suggested by some studies, macrophages influence tumor cells through various mechanisms and have become a new research hotspot in immunotherapy research (45, 46, 64), and researchers have found that certain cytokines (56) secreted by macrophages or modified macrophages can be used to kill tumor cells. In recent years, a variety of recognition mechanisms have been discovered, and related targeted therapies, such as the application of antibodies or inhibitors (43, 71), genetic modification (46), and adoptive transfer of immune cells, are under in-depth research. In summary, macrophages are promising in terms of tumor-targeted therapy, as several kinds of therapy has been applied, but the technology is still immature at present, and current researches are limited because cancer still cannot be completely cured. Thus, quite a few unknown molecular mechanisms may play a vitally important role in the regulation of tumor growth and development, and some potential targets need more research and attention. Thus, it is necessary to investigate the communication of macrophages and tumor cells a bit deeper in further studies.

## Author Contributions

All authors wrote the manuscript and JZ designed the figures. ZT and CL collected the related references. SG and YF edited the manuscript. XZ provided guidance and revised this manuscript. All authors approved the final manuscript.

### Conflict of Interest

The authors declare that the research was conducted in the absence of any commercial or financial relationships that could be construed as a potential conflict of interest.

## Abbreviations

TAMs: tumor-associated macrophages
M1: M1 macrophages
M2: M2 macrophages
LPS: lipopolysaccharides
CCL2: chemokine (C-C motif) ligand 2
STAT3: signal transducer and activator of transcription 3
EMT: epithelial-mesenchymal transition
Bcl-2: B-cell lymphoma-2
JAK: janus kinase
ELMO1: engulfment and cell motility 1
IBC: inflammatory breast cancer
GRO: growth-related oncogene
TGF-β: growth factor-β
PGE2: prostaglandin E2
MMP-7: matrix metalloproteinase-7
TLR4: Toll-like receptor 4
CIP2A: cancerous inhibitor of PP2A
PI3K: phosphatidylinositide 3-kinases
PD-1: programmed cell death protein
PD-L1: programmed cell death-ligand 1
NK: natural killer
CD47: cluster of differentiation 47
SIRPα: signal regulatory protein alpha
ITIM: immunoreceptor tyrosine-based inhibitory motif
MHC: major histocompatibility complex
LILRB1: leukocyte immunoglobulin like receptor subfamily B member 1
Siglc-10: sialic-acid-binding Ig-like lectin 10
ApoE: apolipoprotein E
PDAC: pancreatic ductal adenocarcinoma
MDE: macrophage-derived exosomes
PrCR: programmed cell removal
Btk: Bruton's tyrosine kinase
CRT: calreticulin
MDP: muramyl dipeptide
BCG: bacilli calmette-guerin
sTn: Sialyl-Tn
tEVs: tumor-derived extracellular vesicles
MCP-1: monocyte chemotactic protein-1
CSF-1: colony stimulating factor-1
CREB: cAMP response element binding protein
GCN2: general control nonderepressible 2
MDSCs: myeloid-derived suppressor cells
ATF4: activating transcription factor 4
HAC: an engineered small protein which can block human PD-L1
QPCTL: glutaminyl-peptide cyclotransferase-like protein
ADCP: antibody-dependent cell phagocytosis
AZA: azacytidine
DFMO: difluoromethylornithine
iSNAPS: integrated sensing and activating protein.

## References

[1] Haniffa M Bigley V Collin M. Human mononuclear phagocyte system reunited. Semin Cell Dev Biol. (2015) 41:59–69. 10.1016/j.semcdb.2015.05.004 25986054

[2] Yona S Gordon S. From the reticuloendothelial to mononuclear phagocyte system - the unaccounted years. Front Immunol. (2015) 6:328. 10.3389/fimmu.2015.00328 26191061 PMC4486871

[3] Gordon S Taylor PR. Monocyte and macrophage heterogeneity. Nat Rev Immunol. (2005) 5:953. 10.1038/nri1733 16322748

[4] Santoni M Bracarda S Nabissi M Massari F Conti A Bria E. CXC and CC chemokines as angiogenic modulators in nonhaematological tumors. Bio Med Res Int. (2014) 2014:768758. 10.1155/2014/768758 24971349 PMC4058128

[5] Ginhoux F Jung S. Monocytes and macrophages: developmental pathways and tissue homeostasis. Nat Rev Immunol. (2014) 14:392–404. 10.1038/nri3671 24854589

[6] Olingy CE Dinh HQ Hedrick CC. Monocyte heterogeneity and functions in cancer. J Leukoc Biol. (2019) 106:309–22. 10.1002/JLB.4RI0818-311R 30776148 PMC6658332

[7] Varol C Mildner A Jung S. Macrophages: development and tissue specialization. Annu Rev Immunol. (2015) 33:643–75. 10.1146/annurev-immunol-032414-112220 25861979

[8] Luke CD Jenkins SJ Allen JE Taylor PR Tissue-resident macrophages. Nat Immunol. (2013) 14:986–95. 10.1038/ni.2705 24048120 PMC4045180

[9] Fleetwood AJ Lawrence T Hamilton JA Cook AD. Granulocyte-macrophage colony-stimulating factor (CSF) and macrophage CSF-dependent macrophage phenotypes display differences in cytokine profiles and transcription factor activities: implications for CSF blockade in inflammation. J Immunol. (2007) 178:5245–52. 10.4049/jimmunol.178.8.5245 17404308

[10] Arnold CE Whyte CS Gordon P Barker RN Rees AJ Wilson HM. A critical role for suppressor of cytokine signalling 3 in promoting M1 macrophage activation and function *in vitro* and *in vivo*. Immunology. (2014) 141:96–110. 10.1111/imm.12173 24088176 PMC3893853

[11] Sica A Larghi P Mancino A Rubino L Porta C Totaro MG. Macrophage polarization in tumour progression. Semin Cancer Biol. (2008) 18:349–55. 10.1016/j.semcancer.2008.03.004 18467122

[12] Jenkins SJ Ruckerl D Thomas GD Hewitson JP Duncan S Brombacher F. IL-4 directly signals tissue-resident macrophages to proliferate beyond homeostatic levels controlled by CSF-1. J Exp Med. (2013) 210:2477–91. 10.1084/jem.20121999 24101381 PMC3804948

[13] Murray PJ. Macrophage polarization. Annu Rev Physiol. (2017) 79:541–66. 10.1146/annurev-physiol-022516-034339 27813830

[14] Chávez-Galán L Olleros ML Vesin D Garcia I. Much more than M1 and M2 macrophages, there are also CD169^+^ and TCR^+^ macrophages. Front Immunol. (2015) 6:263. 10.3389/fimmu.2015.00263 26074923 PMC4443739

[15] Crocker PR Gordon S. Properties and distribution of a lectin-like hemagglutinin differentially expressed by murine stromal tissue macrophages. J Exp Med. (1986) 164:1862–75. 10.1084/jem.164.6.1862 3783087 PMC2188478

[16] Martínez-Pomares L Kosco-Vilbois M Darley E Tree P Herren S Bonnefoy JY. Fc chimeric protein containing the cysteine-rich domain of the murine mannose receptor binds to macrophages from splenic marginal zone and lymph node subcapsular sinus and to germinal centers. J Exp Med. (1996) 184:1927–37. 10.1084/jem.184.5.1927 8920880 PMC2192889

[17] Martinez-Pomares L Gordon S. CD169^+^ macrophages at the crossroads of antigen presentation. Trends Immunol. (2012) 33:66–70. 10.1016/j.it.2011.11.001 22192781

[18] Puellmann K Kaminski WE Vogel M Nebe CT Schroeder J Wolf H. A variable immunoreceptor in a subpopulation of human neutrophils. Proc Natl Acad Sci USA. (2006) 103:14441–6. 10.1073/pnas.0603406103 16983085 PMC1599981

[19] Kaminski WE Beham AW Kzhyshkowska J Gratchev A Puellmann K. On the horizon: flexible immune recognition outside lymphocytes. Immunobiology. (2013) 218:418–26. 10.1016/j.imbio.2012.05.024 22749215

[20] Morrison C. Immuno-oncologists eye up macrophage targets. Nat Rev Drug Discov. (2016) 15:373–4. 10.1038/nrd.2016.111 27245386

[21] Azizi E Carr AJ Plitas G Cornish AE Konopacki C Prabhakaran S. Single-cell map of diverse immune phenotypes in the breast tumor microenvironment. Cell. (2018) 174:1293–308 e1236. 10.1016/j.cell.2018.05.060 29961579 PMC6348010

[22] Qiu SQ Waaijer SJH Zwager MC de Vries EGE van der Vegt B Schröder CP Tumor-associated macrophages in breast cancer: innocent bystander or important player? Cancer Treat Rev. (2018) 70:178–89. 10.1016/j.ctrv.2018.08.010 30227299

[23] Mantovani A Savino B Locati M Zammataro L Allavena P Bonecchi R. The chemokine system in cancer biology and therapy. Cytokine Growth Factor Rev. (2010) 21:27–39. 10.1016/j.cytogfr.2009.11.007 20004131

[24] Myers KV Amend SR Pienta KJ. Targeting Tyro3, Axl and MerTK (TAM receptors): implications for macrophages in the tumor microenvironment. Cancer. (2019) 18:94. 10.1186/s12943-019-1022-2 31088471 PMC6515593

[25] Murray PJ Allen JE Biswas SK Fisher EA Gilroy DW Goerdt S. Macrophage activation and polarization: nomenclature and experimental guidelines. Immunity. (2014) 41:14–20. 10.1016/j.immuni.2014.06.008 25035950 PMC4123412

[26] Di Caro G Cortese N Castino GF Grizzi F Gavazzi F Ridolfi C Dual prognostic significance of tumour-associated macrophages in human pancreatic adenocarcinoma treated or untreated with chemotherapy. Gut. (2016) 65:1710–20. 10.1136/gutjnl-2015-309193 26156960

[27] Salaroglio IC Kopecka J Napoli F Pradotto M Maletta F Costardi L Potential diagnostic and prognostic role of micro-environment in malignant pleural mesothelioma. J Thorac Oncol. (2019) 14:1458–71. 10.1016/j.jtho.2019.03.029 31078776

[28] Gao S Hu J Wu X Liang Z. PMA treated THP-1-derived-IL-6 promotes EMT of SW48 through STAT3/ERK-dependent activation of Wnt/β-catenin signaling pathway. Biomed Pharmacother. (2018) 108:618–24. 10.1016/j.biopha.2018.09.067 30243096

[29] Yadav A Kumar B Datta J Teknos TN Kumar P. IL-6 promotes head and neck tumor metastasis by inducing epithelial-mesenchymal transition via the JAK-STAT3-SNAIL signaling pathway. Mol Cancer Res. (2011) 9:1658–67. 10.1158/1541-7786.MCR-11-0271 21976712 PMC3243808

[30] Leu CM Wong FH Chang C Huang SF Hu CP. Interleukin-6 acts as an antiapoptotic factor in human esophageal carcinoma cells through the activation of both STAT3 and mitogen-activated protein kinase pathways. Oncogene. (2003) 22:7809–18. 10.1038/sj.onc.1207084 14586407

[31] Williams CB Yeh ES Soloff AC. Tumor-associated macrophages: unwitting accomplices in breast cancer malignancy. NPJ Breast Cancer. (2016) 2:15025. 10.1038/npjbcancer.2015.25 26998515 PMC4794275

[32] Sanmamed MF Perez-Gracia JL Schalper KA Fusco JP Gonzalez A Rodriguez-Ruiz ME. Changes in serum interleukin-8 (IL-8) levels reflect and predict response to anti-PD-1 treatment in melanoma and non-small-cell lung cancer patients. Ann Oncol. (2017) 28:1988–95. 10.1093/annonc/mdx190 28595336 PMC5834104

[33] Shao N Lu Z Zhang Y Wang M Li W Hu Z. Interleukin-8 upregulates integrin beta3 expression and promotes estrogen receptor-negative breast cancer cell invasion by activating the PI3K/Akt/NF-kappaB pathway. Cancer Lett. (2015) 364:165–72. 10.1016/j.canlet.2015.05.009 25979232

[34] Deng J Liang H Zhang R Sun D Pan Y Liu Y. STAT3 is associated with lymph node metastasis in gastric cancer. Tumour Biol. (2013) 34:2791–800. 10.1007/s13277-013-0837-5 23824569

[35] Valeta-Magara A Gadi A Volta V Walters B Arju R Giashuddin S Inflammatory breast cancer promotes development of M2 tumor-associated macrophages and cancer mesenchymal cells through a complex cytokine network. Cancer Res. (2019) 79:3360–71. 10.1158/0008-5472.CAN-17-2158 31043378 PMC7331114

[36] Ouyang W O'Garra A. IL-10 family cytokines IL-10 and IL-22: from basic science to clinical translation. Immunity. (2019) 50:871–91. 10.1016/j.immuni.2019.03.020 30995504

[37] Banerjee S Halder K Bose A Bhattacharya P Gupta G Karmahapatra S. TLR signaling-mediated differential histone modification at IL-10 and IL-12 promoter region leads to functional impairments in tumor-associated macrophages. Carcinogenesis. (2011) 32:1789–97. 10.1093/carcin/bgr208 21926109

[38] Liu CY Xu JY Shi XY Huang W Ruan TY Xie P. M2-polarized tumor-associated macrophages promoted epithelial-mesenchymal transition in pancreatic cancer cells, partially through TLR4/IL-10 signaling pathway. Lab Invest. (2013) 93:844–54. 10.1038/labinvest.2013.69 23752129

[39] Sung WW Wang YC Lin PL Cheng YW Chen CY Wu TC. IL-10 promotes tumor aggressiveness via upregulation of CIP2A transcription in lung adenocarcinoma. Clin Cancer Res. (2013) 19:4092–103. 10.1158/1078-0432.CCR-12-3439 23743567

[40] Sato T Terai M Tamura Y Alexeev V Mastrangelo MJ Selvan SR. Interleukin 10 in the tumor microenvironment: a target for anticancer immunotherapy. Immunol Res. (2011) 51:170–82. 10.1007/s12026-011-8262-6 22139852

[41] Boussiotis VA Chatterjee P Li L. Biochemical signaling of PD-1 on T cells and its functional implications. Cancer J. (2014) 20:265–71. 10.1097/PPO.0000000000000059 25098287 PMC4151049

[42] Yu GT Bu LL Huang CF Zhang WF Chen WJ Gutkind JS. PD-1 blockade attenuates immunosuppressive myeloid cells due to inhibition of CD47/SIRPalpha axis in HPV negative head and neck squamous cell carcinoma. Oncotarget. (2015) 6:42067–80. 10.18632/oncotarget.5955 26573233 PMC4747210

[43] Gordon SR Maute RL Dulken BW Hutter G George BM McCracken MN. PD-1 expression by tumour-associated macrophages inhibits phagocytosis and tumour immunity. Nature. (2017) 545:495. 10.1038/nature22396 28514441 PMC5931375

[44] Katsuya Y Horinouchi H Asao T Kitahara S Goto Y Kanda S. Expression of programmed death 1 (PD-1) and its ligand (PD-L1) in thymic epithelial tumors: impact on treatment efficacy and alteration in expression after chemotherapy. Lung Cancer. (2016) 99:4–10. 10.1016/j.lungcan.2016.05.007 27565906

[45] Chao MP Weissman IL Majeti R. The CD47-SIRPalpha pathway in cancer immune evasion and potential therapeutic implications. Curr Opin Immunol. (2012) 24:225–32. 10.1016/j.coi.2012.01.010 22310103 PMC3319521

[46] Barkal AA Weiskopf K Kao KS Gordon SR Rosental B Yiu YY. Engagement of MHC class I by the inhibitory receptor LILRB1 suppresses macrophages and is a target of cancer immunotherapy. Nat Immunol. (2018) 19:76–84. 10.1038/s41590-017-0004-z 29180808 PMC5832354

[47] Barkal AA Brewer RE Markovic M Kowarsky M Barkal SA Zaro BW. CD24 signalling through macrophage Siglec-10 is a target for cancer immunotherapy. Nature. (2019) 572:392–6. 10.1038/s41586-019-1456-0 31367043 PMC6697206

[48] Milane L Singh A Mattheolabakis G Suresh M Amiji MM. Exosome mediated communication within the tumor microenvironment. J Control Release. (2015) 219:278–94. 10.1016/j.jconrel.2015.06.029 26143224

[49] Syn N Wang L Sethi G Thiery JP Goh BC. Exosome-mediated metastasis: from epithelial-mesenchymal transition to escape from immunosurveillance. Trends Pharmacol Sci. (2016) 37:606–17. 10.1016/j.tips.2016.04.006 27157716

[50] Yeung OW Lo CM Ling CC Qi X Geng W Li CX. Alternatively activated (M2) macrophages promote tumour growth and invasiveness in hepatocellular carcinoma. J Immunol. (2015) 62:607–16. 10.1016/j.jhep.2014.10.029 25450711

[51] Zheng P Luo Q Wang W Li J Wang T Wang P. Tumor-associated macrophages-derived exosomes promote the migration of gastric cancer cells by transfer of functional Apolipoprotein E. Cell Death Dis. (2018) 9:434. 10.1038/s41419-018-0465-5 29567987 PMC5864742

[52] Binenbaum Y Fridman E Yaari Z Milman N Schroeder A Ben David G. Transfer of miRNA in macrophage-derived exosomes induces drug resistance in pancreatic adenocarcinoma. Cancer Res. (2018) 78:5287–99. 10.1158/0008-5472.CAN-18-0124 30042153

[53] Byrne JC Ní Gabhann J Stacey KB Coffey BM McCarthy E Thomas W. Bruton's tyrosine kinase is required for apoptotic cell uptake via regulating the phosphorylation and localization of calreticulin. J Immunol. (2013) 190:5207–15. 10.4049/jimmunol.1300057 23596312

[54] Ogden CA deCathelineau A Hoffmann PR Bratton D Ghebrehiwet B Fadok VA. C1q and mannose binding lectin engagement of cell surface calreticulin and CD91 initiates macropinocytosis and uptake of apoptotic cells. J Exp Med. (2001) 194:781–95. 10.1084/jem.194.6.781 11560994 PMC2195958

[55] Holla S Ghorpade DS Singh V Bansal K Balaji KN. *Mycobacterium bovis* BCG promotes tumor cell survival from tumor necrosis factor-alpha-induced apoptosis. Mol Cancer. (2014) 13:210. 10.1186/1476-4598-13-210 25208737 PMC4174669

[56] Wang H Zhang L Yang L Liu C Zhang Q Zhang L. Targeting macrophage anti-tumor activity to suppress melanoma progression. Oncotarget. (2017) 8:18486–96. 10.18632/oncotarget.14474 28060744 PMC5392344

[57] Moran I Grootveld AK Nguyen A Phan TG. Subcapsular sinus macrophages: the seat of innate and adaptive memory in murine lymph nodes. Trends Immunol. (2019) 40:35–48. 10.1016/j.it.2018.11.004 30502023

[58] Colombo M Raposo G Théry C. Biogenesis, secretion, and intercellular interactions of exosomes and other extracellular vesicles. Annu Rev Cell Dev Biol. (2014) 30:255–89. 10.1146/annurev-cellbio-101512-122326 25288114

[59] Pucci F Garris C Lai CP Newton A Pfirschke C Engblom C. SCS macrophages suppress melanoma by restricting tumor-derived vesicle–B cell interactions. Science. (2016) 352:242–6. 10.1126/science.aaf1328 26989197 PMC4960636

[60] Tang CH Tsai CC. CCL2 increases MMP-9 expression and cell motility in human chondrosarcoma cells via the Ras/Raf/MEK/ERK/NF-κB signaling pathway. Biochem Pharmacol. (2012) 83:335–44. 10.1016/j.bcp.2011.11.013 22138288

[61] Tsagozis P Eriksson F Pisa P. Zoledronic acid modulates antitumoral responses of prostate cancer-tumor associated macrophages. Cancer Immunol Immunother. (2008) 57:1451–9. 10.1007/s00262-008-0482-9 18297280 PMC11030129

[62] Borghese C Cattaruzza L Pivetta E Normanno N De Luca A Mazzucato M. Gefitinib inhibits the cross-talk between mesenchymal stem cells and prostate cancer cells leading to tumor cell proliferation and inhibition of docetaxel activity. J Cell Biochem. (2013) 114:1135–44. 10.1002/jcb.24456 23192362

[63] Mok S Koya RC Tsui C Xu J Robert L Wu L. Inhibition of CSF-1 receptor improves the antitumor efficacy of adoptive cell transfer immunotherapy. Cancer Res. (2014) 74:153–61. 10.1158/0008-5472.CAN-13-1816 24247719 PMC3947337

[64] Tymoszuk P Evens H Marzola V Wachowicz K Wasmer MH Datta S. *In situ* proliferation contributes to accumulation of tumor-associated macrophages in spontaneous mammary tumors. Eur J Immunol. (2014) 44:2247–62. 10.1002/eji.201344304 24796276

[65] Zhu Y Knolhoff BL Meyer MA Nywening TM West BL Luo J. CSF1/CSF1R blockade reprograms tumor-infiltrating macrophages and improves response to T-cell checkpoint immunotherapy in pancreatic cancer models. Cancer Res. (2014) 74:5057–69. 10.1158/0008-5472.CAN-13-3723 25082815 PMC4182950

[66] Cassetta L Pollard JW. Targeting macrophages: therapeutic approaches in cancer. Nat Rev Drug Discov. (2018) 17:887–904. 10.1038/nrd.2018.169 30361552

[67] Su L Zhang J Xu H Wang Y Chu Y Liu R. Differential expression of CXCR4 is associated with the metastatic potential of human non-small cell lung cancer cells. Clin Cancer Res. (2005) 11:8273–80. 10.1158/1078-0432.CCR-05-0537 16322285

[68] Halaby MJ Hezaveh K Lamorte S Ciudad MT Kloetgen A MacLeod BL. GCN2 drives macrophage and MDSC function and immunosuppression in the tumor microenvironment. Sci Immunol. (2019) 4:eaax8189. 10.1126/sciimmunol.aax8189 31836669 PMC7201901

[69] Topalian SL Hodi FS Brahmer JR Gettinger SN Smith DC McDermott DF. Safety, activity, and immune correlates of anti–PD-1 antibody in cancer. N Engl J Med. (2012) 366:2443–54. 10.1056/NEJMoa1200690 22658127 PMC3544539

[70] Chao MP Majeti R Weissman IL. Programmed cell removal: a new obstacle in the road to developing cancer. Nat Rev Cancer. (2011) 12:58–67. 10.1038/nrc3171 22158022

[71] Oldenborg PA Gresham HD Chen Y Izui S Lindberg FP. Lethal autoimmune hemolytic anemia in CD47-deficient nonobese diabetic (NOD) mice. Blood. (2002) 99:3500–4. 10.1182/blood.V99.10.3500 11986200

[72] Weiskopf K Ring AM Ho CC Volkmer JP Levin AM Volkmer AK. Engineered SIRPα variants as immunotherapeutic adjuvants to anticancer antibodies. Science. (2013) 341:88–91. 10.1126/science.1238856 23722425 PMC3810306

[73] Liu J Wang L Zhao F Tseng S Narayanan C Shura L. Pre-clinical development of a humanized anti-CD47 antibody with anti-cancer therapeutic potential. PLoS ONE. (2015) 10:1–23. 10.1371/journal.pone.0137345 26390038 PMC4577081

[74] Chao MP Alizadeh AA Tang C Jan M Weissman-Tsukamoto R Zhao F. Therapeutic antibody targeting of CD47 eliminates human acute lymphoblastic leukemia. Cancer Res. (2011) 71:1374–84. 10.1158/0008-5472.CAN-10-2238 21177380 PMC3041855

[75] Horrigan SK Reproducibility Project: Cancer Biology. Replication study: the CD47-signal regulatory protein alpha (SIRPa) interaction is a therapeutic target for human solid tumors. Elife. (2017) 6:e18173. 10.7554/eLife.18173 28100392 PMC5245970

[76] Advani R Flinn I Popplewell L Forero A Bartlett NL Ghosh N. cd47 blockade by hu5f9-g4 and rituximab in non-hodgkin's lymphoma. N Engl J Med. (2018) 379:1711–21. 10.1056/NEJMoa1807315 30380386 PMC8058634

[77] Logtenberg MEW Jansen JHM Raaben M Toebes M Franke K Brandsma AM. Glutaminyl cyclase is an enzymatic modifier of the CD47- SIRPα axis and a target for cancer immunotherapy. Nat Med. (2019) 25:612–9. 10.1038/s41591-019-0356-z 30833751 PMC7025889

[78] Alvey CM Spinler KR Irianto J Pfeifer CR Hayes B Xia Y. SIRPA-inhibited, marrow-derived macrophages engorge, accumulate, and differentiate in antibody-targeted regression of solid tumors. Curr Biol. (2017) 27:2065–77. e2066. 10.1016/j.cub.2017.06.005 28669759 PMC5846676

[79] Ring NG Herndler-Brandstetter D Weiskopf K Shan L Volkmer JP George BM. Anti-SIRPα antibody immunotherapy enhances neutrophil and macrophage antitumor activity. Proc Natl Acad Sci USA. (2017) 114:E10578–85. 10.1073/pnas.1710877114 29158380 PMC5724266

[80] Overdijk MB Verploegen S Bögels M van Egmond M Lammerts van Bueren JJ Mutis T. Antibody-mediated phagocytosis contributes to the anti-tumor activity of the therapeutic antibody daratumumab in lymphoma and multiple myeloma. MAbs. (2015) 7:311–21. 10.1080/19420862.2015.1007813 25760767 PMC4622648

[81] Banerjee S Halder K Ghosh S Bose A Majumdar S. The combination of a novel immunomodulator with a regulatory T cell suppressing antibody (DTA-1) regress advanced stage B16F10 solid tumor by repolarizing tumor associated macrophages *in situ*. Oncoimmunology. (2015) 4:e995559. 10.1080/2162402X.2014.995559 25949923 PMC4404885

[82] Travers M Brown SM Dunworth M Holbert CE Wiehagen KR Bachman KE. DFMO and 5-azacytidine increase M1 macrophages in the tumor microenvironment of murine ovarian cancer. Cancer Res. (2019) 79:3445–54. 10.1158/1538-7445.AM2019-2805 31088836 PMC6606334

[83] Ramesh A Kumar S Nandi D Kulkarni A. CSF1R- and SHP2-inhibitor-loaded nanoparticles enhance cytotoxic activity and phagocytosis in tumor-associated macrophages. Adv Mater. (2019) 31:e1904364. 10.1002/adma.201904364 31659802

[84] Sun J Lei L Tsai CM Wang Y Shi Y Ouyang M. Engineered proteins with sensing and activating modules for automated reprogramming of cellular functions. Nat Commun. (2017) 8:477. 10.1038/s41467-017-00569-6 28883531 PMC5589908

[85] Pienta KJ Bradley D. Mechanisms underlying the development of androgen-independent prostate cancer. Clin Cancer Res. (2006) 12:1665–71. 10.1158/1078-0432.CCR-06-0067 16551847

[86] Okazawa H Motegi S Ohyama N Ohnishi H Tomizawa T Kaneko Y. Negative regulation of phagocytosis in macrophages by the CD47-SHPS-1 system. J Immunol. (2005) 174:2004–11. 10.4049/jimmunol.174.4.2004 15699129

